# Potential of Nano-Gum Arabic on the Physical, Mechanical, Adhesive, Optical, and Biological Performance of Glass Ionomer Cement: A Comprehensive In Vitro Study

**DOI:** 10.1186/s12903-026-08567-1

**Published:** 2026-06-09

**Authors:** Marwa Beleidy, Soha A. Hassan, Rania Rashad Omar Taha, Yousra Nashaat, Yasmine Alaa El-din

**Affiliations:** 1 Fixed Prosthodontics Department, Faculty of Dentistry, October 6 University, Giza, Egypt; 2 Faculty of Applied Health Sciences Technology, October 6th University, Giza, Egypt; 3 Conservastive Dentistry Department, Faculty of Dentistry, Suez University, Suez, Egypt; 4 Endodontics Department, Faculty of Dentistry, October 6 University, Giza, Egypt; 5 Oral and Maxillofacial Pathology Department, Faculty of Dentistry, October 6 University, Giza, Egypt

**Keywords:** Glass ionomer cement, Nano-Gum Arabic; water sorption and solubility, Diametral tensile strength, Shear bond strength, Translucency parameter, Biocompatibility

## Abstract

**Background:**

Glass Ionomer Cements (GICs) have limited strength, esthetics, and bioactivity. This study aimed to develop a nanoformulation of Gum Arabic (GA) and incorporate it into a commonly used GIC material to evaluate its effects on the cement’s physical, mechanical, adhesive, optical, and biological properties.

**Methods:**

GA was oxidized to introduce active functional groups. The resulting solution was atomized using a nanospray dryer to obtain nano-Gum Arabic (nano-GA) powder. Dynamic light scattering (DLS) analysis confirmed the nanoparticle size distribution. Nano-GA was incorporated into GIC (Medicem; Promedica, Dental Material GmbH, Neumuenster, Germany) at concentrations of 0.5, 1.0, 2.0, 4.0, 8.0, and 16.0 wt%. The effects of nano-GA addition on film thickness, water solubility (Wsol) and sorption (Wsp), diametral tensile strength (DTS), shear bond strength (SBS) to enamel and dentin, translucency parameter (TP), and contrast ratio (CR) were evaluated. Biological assessments were also conducted, including cytotoxicity, genotoxicity, and cell viability analyses. Data were statistically analyzed using one-way ANOVA and post hoc tests at a significance level of *p* < 0.05.

**Results:**

The 8.0 wt% group exhibited the highest film thickness among all tested formulations, while the 2.0 wt% concentration demonstrated the lowest Wsol. Increasing nano-GA content up to 16 wt% reduced Wsp compared to the control (*p* < 0.05). No significant differences in DTS were observed among the 0.5, 1.0, and 4.0 wt% groups. Regarding adhesive performance, 2.0 wt% showed superior SBS to enamel, whereas the 1.0 wt% formulation achieved higher SBS to dentin. Optical assessments revealed that the 16.0 wt% group had the highest TP values, while the CR decreased with increasing nano-GA content. Biological evaluation confirmed that nano-GA addition up to 4.0 wt% exhibited no cytotoxic or genotoxic effects; however, cell cycle analysis indicated a slight increase in apoptosis at 4.0 wt%, whereas the G0/G1 phase percentage was highest in the 0 wt% group.

**Conclusions:**

Incorporating nano-GA into conventional GIC improved its physical, mechanical, adhesive, optical, and biological properties in a concentration-dependent manner. Optimal performance was achieved at 0.5-2.0 wt% nano-GA, enhancing strength, adhesion, and stability without affecting translucency or biocompatibility. Nano-GA up to 4.0 wt% was biocompatible, while higher concentrations induced cytotoxic effects. Thus, nano-GA is a promising additive for improving GIC performance at optimized concentrations.

## Background

Adequate working and setting times are essential for any luting material used in fixed dental prosthesis (FDP) cementation. In addition, an ideal luting agent should exhibit low solubility, strong adhesion, biocompatibility, anti-cariogenic properties, good marginal adaptation, and sufficient mechanical strength [1]. Although self-adhesive resin cements show promise as modern luting materials, they lack the anti-cariogenic properties of glass ionomer cements (GICs) and exhibit moisture sensitivity [2]. Reported failure rates of resin cements in fixed prosthodontics, ranging from 4.1% for fiber-reinforced composites to 11.7% for all-ceramic restorations, highlight persistent issues such as debonding, delamination, and framework fracture [3, 4]. Conversely, GICs offer distinct advantages, including biocompatibility, chemical bonding to tooth structure, fluoride release, and protection against secondary caries [5]. However, their inherently low mechanical strength remains a significant limitation, often leading to clinical failure due to inadequate stress transfer from FDPs to the supporting tooth structure [6].

Several previous and ongoing advancements aim to incorporate filler components in the GIC powder, such as silver-amalgam particles, zirconia, glass fibers, hydroxyapatite, glass surface pretreatment, and glass composition modification [7, 8]. Furthermore, nanotechnology in dentistry has attracted many researchers’ interest in the last few years since incorporating nanoparticles (NPs) exhibits unique physical and chemical properties necessary in many dental materials [9].

Natural polymers such as Gum Arabic (GA) have gained increasing attention as bio-based additives for enhancing the properties of GICs. GA, also known as Gum Acacia, is a naturally derived, non-toxic, and biocompatible polysaccharide obtained from the hardened exudates of Acacia senegal and Acacia seyal trees [10–12]. It exhibits antibacterial activity and has been widely utilized as a bioactive carrier and binder in pharmaceutical and biomedical applications owing to its excellent film-forming and stabilizing capabilities. Chemically, GA is primarily composed of arabinose and galactose units with abundant hydroxyl groups that can be chemically modified to enhance reactivity. Oxidation of GA introduces carboxyl and carbonyl functional groups, increasing its potential for ionic and hydrogen bonding with glass particles and the polyacid matrix of GICs. Such interactions are anticipated to improve interfacial adhesion, mechanical integrity, and biocompatibility. Previous investigations have demonstrated that oxidized or nano-structured GA can effectively reinforce dental and polymeric composites, enhancing structural stability, reducing solubility, and minimizing crack propagation within GICs [13–16].

Some studies have reported variations in the cytotoxic responses of GICs depending on testing conditions [17–21]. These differences have been attributed to several factors, including ion leaching, variations in chemical composition, and oxidative stress induced by trace amounts of aluminum or iron ions released from the glass matrix [22, 23]. Other investigations have linked cytotoxic effects to the low initial pH during setting and the release of acidic components, which may be influenced by nano-GA incorporation [24]. Moreover, the cytotoxic response of GICs appears to be time-dependent, likely due to transient acidic shock to surrounding cells immediately after material placement [25]. It has also been proposed that formulation differences among commercial GICs contribute to inconsistent biological responses reported in the literature [26]. Incorporation of bioactive natural additives, such as nano-GA, may therefore enhance the biological performance of GICs by mitigating these cytotoxic effects.

This study aimed to evaluate the effect of incorporating oxidized nano-GA into a conventional GIC at several different concentrations on the cement’s physical properties (film thickness, water sorption-Wsp and solubility-Wsol), mechanical performance (diametral tensile strength-DTS), and adhesive behavior (shear bond strength-SBS to enamel and dentin). Furthermore, optical properties (translucency parameter-TP and contrast ratio-CR) and biological responses (cytotoxicity, genotoxicity, and cell analysis) were examined to determine the optimal concentration for improving GIC performance. The research hypotheses were formulated as follows:

1. The first hypothesis stated that nano-GA addition would not increase the film thickness of GIC beyond clinically acceptable limits while reducing its Wsp and Wsol.
2. The second hypothesis stated that nano-GA incorporation would enhance the mechanical and adhesive performance of GIC, including DTS and SBS to enamel and dentin.
3. The third hypothesis stated that nano-GA incorporation would not affect the optical performance of GIC, including TP and CR.
4. The fourth hypothesis stated that nano-GA incorporation would not affect the biological response of GIC, maintaining cytotoxicity and genotoxicity levels comparable to the unmodified control.

## Methods

### Sample size calculation

A 0.05 alpha (α) level, 0.2 beta (β) corresponding to 80% statistical power, and a 0.658 effect size (f) derived from Singer et al.‘s research, considering SBS as the primary outcome, were utilized for power analysis conduction [27]. The minimum total requisite sample size (n) was determined to be six specimens per group. Sample size was calculated using a sample size software (G*Power; Version 3.1.9.2, HHUD, Germany).

### Oxidation and nanoformulation of GA

Five grams of GA (Organic CLEAR Acacia Powder, 365 by Whole Foods Market, London, UK) was incorporated into 100 ml of Millipore water in a 500 ml round-bottom flask and homogenized gently at 11,000 rpm for 30 min at 70 °C. Subsequently, 150 ml of 30% hydrogen peroxide was introduced while stirring for 10 min, then 10 mg of ferrous sulfate was added as a catalyst. The reaction was elevated to 100 °C for 120 min under condensation conditions [28].

After completion of the reaction, the oxidized GA solution was subjected to nanospray drying using a laboratory nanospray dryer (B-90, BÜCHI Labortechnik AG, Flawil, Switzerland) to obtain fine, free-flowing nanopowder. The collected nano-Gum Arabic (nano-GA) was characterized to confirm its nanoscale formation and colloidal stability. Dynamic light scattering (DLS) analysis (Zetasizer Nano ZS, Malvern Instruments, Malvern, UK) was employed to determine the average particle size and size distribution of the nano-GA. Measurements were performed at 25 °C after appropriate dilution with deionized water to prevent multiple scattering.

### Specimens’ preparation

A commercially available type 1 GIC (Medicem (Promedica, Dental Material GmbH, Neumuenster, Germany) was chosen and provided in a powder-liquid system. Its chemical composition is listed in Table 1. The oxidized nano- GA was incorporated into the GIC powder using a geometric dilution method to ensure uniform nanoparticle distribution and minimize agglomeration [28]. Predetermined amounts of nano-GA (0.5, 1.0, 2.0, 4.0, 8.0, and 16.0 wt%) were accurately weighed using a precision digital microbalance (AS 82/220.R2 PLUS Analytical Balance, 220 g Capacity, 0.001 g Readability Radwag, Radom, Poland) (± 0.0001 g accuracy) and initially mixed with a small portion of GIC powder using a mortar and pestle. Additional GIC powder was gradually added in increments, blending thoroughly at each step. The powder mixture was further homogenized using an amalgamator for 30 s [29]. The modified powders were stored in airtight containers at room temperature until use.

**Table 1:** Chemical composition of the commercial glass ionomer cement used in the current study

Cement	Component	Type	Chemical Composition	Manufacturer
Medicem Conventional GIC	Powder	Fluoroalumino-silicate glass	SiO₂ (45–50%), Al₂O₃ (15–20%), AlF₃ (5–10%), Na₃AlF₆ (5–8%), CaF₂ (5–10%), AlPO₄ (1–3%)	Promedica Dental Material GmbH, Neumünster, Germany
	Liquid	Aqueous solution of polyacrylic acid	Polyacrylic acid (~ 40–45%), tartaric acid (~ 5–10%),	

The control and experimental GIC powders were mixed manually according to the manufacturer’s recommended powder-to-liquid ratio of 3.6:1 (by weight) on a glass slab for 30–45 s until a glossy mixture was obtained.

Specimens were prepared using cylindrical Teflon molds with dimensions standardized for each specific test: 9 × 2 mm for water sorption and solubility, 6 × 3 mm for DTS, 10 × 1 mm for TP and CR, and 7 × 1.5 mm for bioactivity evaluation [30, 31]. The mixture paste was poured into a Teflon mold between two glass plates. Subsequently, digital caliper measurements were taken to ensure the specimens’ dimensions (150 mm Digital Caliper, Mitutoyo Corporation, Kanagawa, Japan).

### Physical properties testing

#### Film thickness assessment

Two flat glass plates (5 mm thickness × 20 mm width × 20 mm length) were joined, and their combined thickness was measured with an accuracy of 0.01 μm, designated as measurement (A). A 0.1 ± 0.05 mL volume of each specimen mixture was positioned on a glass plate. Subsequently, the second glass plate was positioned atop the mixture. A universal testing machine was used to apply a 150 N vertical load to the specimen center for 10 s. After 10 min of the load application, the thickness of the two plates was remeasured using a digital caliper (150 mm Digital Caliper, Mitutoyo Corporation, Kanagawa, Japan), representing measurement (B). The difference between the two measurements (B-A) indicates the film thickness [32]. The average film thickness for each study group was attained through six measurements.

#### Water Sorption (Wsp) and Solubility (Wsol)

After 1 h of specimen preparation, 42 specimens (6 specimens per group) were placed in a desiccator containing silica gel (Merck KGaA, Darmstadt, Germany) for 2 h. Subsequently, they were incubated in an oven at 37 °C for 22 h to achieve a constant mass, with a maximum weight variation of ± 0.0005 g. Specimens were measured using a precision analytical balance (Cubis^®^ II Precision Configurable Lab Balance; Sartorius, Bio-pharma Laboratory, Germany) to determine the initial mass (m1) values. Each specimen was incubated in 25 mL of deionized water at 37 ± 1 °C for 7 days. Subsequently, it was dried and reweighed to determine the mass values following immersion (m2). To obtain the final mass after dehydration, the specimen was dehydrated in an incubator at 37 ± 1 °C for 24 h and weighed (m3). The Wsp and Wsol percentages for each specimen were determined using the equation [33].

$$\mathrm{Wsp}={100}\times\mathrm{(m2-m1)}/\text{m1 and Wsol}={100}\times\mathrm{(1-m3)}/\mathrm{m1}$$

### Mechanical and bonding properties testing

#### Diametral Tensile Strength (DTS) test

After 24 h of storage in distilled water at 37 °C to allow full maturation of the glass ionomer cement, each specimen (6 specimens per group) was positioned with its flat ends parallel to the universal testing machine base plate (Model no. 3369, Instron, Canton, MI, USA) to apply load to the specimens’ diameter. The specimens were subjected to static diametric loading using a stainless-steel rod with a flat end (10 cm width × 5 cm length) mounted in the upper movable compartment of the testing machine (Model 3345; Instron Industrial Products, Norwood, MA, USA), applying a 5 kN load at 1.0 mm/min crosshead speed until failure. Data were recorded (Bluehill Lite Software; Instron, MA, USA), and the DTS was calculated in megapascals (MPa) [28].

#### Shear Bond Strength (SBS) and failure mode assessments

Eighty-four human molar teeth were used in the present study; 6 teeth were allocated to each tested group on enamel and dentine. The tested teeth were periodontally debrided and then preserved in distilled water for use within two weeks [34]. Within polyvinyl chloride (PVC) rings, the tooth crowns were embedded within chemically cured acrylic resin, with the buccal surfaces oriented outwardly. After curing, PVC rings were removed, and the formed cylinders were stored in distilled water for 24 h at 37 °C.

**Fig. 1:**
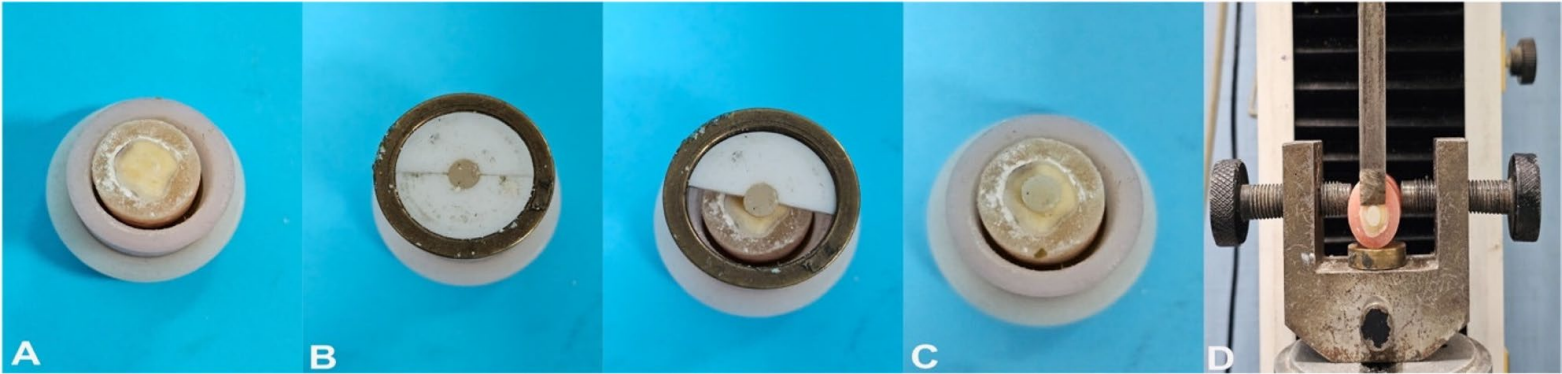
Preparation of a tooth specimen for shear bond strength (SBS) testing. **A** Flattening of the enamel surface to expose 1 mm of dentin. **B** Placement of a split Teflon mold (4 mm diameter × 5 mm height) on the prepared surface and filling with the tested GIC. **C** Set cement. **D** Application of debonding force using a knife-like mandrel during SBS testing

Regarding the enamel group, the mid-buccal region of the embedded crown was sequentially ground using 320 and 600 grit silicon carbide papers under water cooling. The enamel was removed until it had a flat surface and was 1 mm deep into the dentine for the dentine group (Fig. 1A) [35]. A split Teflon mold measuring 4 mm diameter × 5 mm height was fixed on the prepared surfaces and filled with the tested GIC, followed by ISO 29022:2013 (Fig. 1B). After cement setting (Fig. 1C), the specimen was incubated at 100% humidity and 37 °C for 24 h before the SBS assessment.

The SBS was assessed using a computer-controlled materials testing machine (Model 3345; Instron Industrial Products, MA, USA) equipped with a 5 kN load cell, operating at a 0.5 mm/min crosshead speed. A knife-like mandrel was used to apply debonding force on the tested specimen, aligned parallel to the interface between GIC and the specimen (Fig. 1D). The SBS was calculated in MPa using the following formula [34]:

$$\mathrm{SBS}\;\mathrm{(MPa)}=\mathrm{F/A}$$

Where F represents the failure load in Newtons and A denotes the bonded area (r²) in mm².

Each specimen was examined using a USB digital microscope (U500x Digital Microscope, Guangdong, China) with a resolution of 3 megapixels, positioned vertically at a distance of 2.5 cm from the specimens, employing a fixed magnification of 35× to examine the specimens’ fracture surface (debonded surface). Illumination was provided by eight adjustable LED lamps, with a color index nearing 95%. The images were captured (ImageJ 1.43U; National Institute of Health, MD, USA) at a resolution of 1280 × 1024 pixels. The failure modes were classified as follows: (1) adhesive failure, characterized by a fracture occurring entirely within the GIC and enamel/dentine interface, with no GIC remaining on the enamel/dentine surface; (2) cohesive failure, defined by a fracture occurring within the glass-ionomer or enamel/dentine; and (3) mixed failure, identified when a combination of both failure modes was present, with the fracture extending beyond the GIC or enamel/dentine interface [35].

### Optical properties testing

#### Translucency Parameter (TP) and Contrast Ratio (CR) testing

**Fig. 2:**
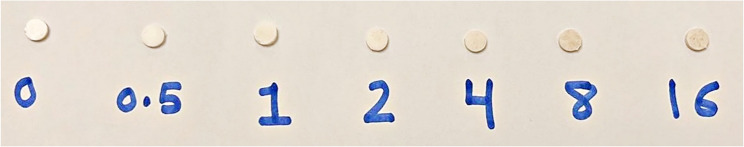
Disk-shaped study specimens of glass ionomer cement (GIC) with varying concentrations of nano-Gum Arabic (nano-GA): 0%, 0.5%, 1.0%, 2.0%, 4.0%, 8.0%, and 16% by weight

After mixing and packing the cement into a mold, the entire assembly was maintained for 1 h at 37 °C with a relative humidity of at least 30%. Subsequently, the cement specimens (6 specimens in each group) were removed from the molds. Then, it was maintained in distilled water at 37 °C for 7 days. Only specimens with a thickness ranging from 0.9 mm to 1.1 mm were utilized in the investigation (Fig. 2) [36].

The TP and CR of the GICs were determined by measuring the specimens with a Reflective Spectrophotometer (Model RM200QC, X-Rite, Neu-Isenburg, Germany). The aperture diameter was configured to 4 mm, and the specimens were precisely aligned at the middle of the measurement port and maintained in the same orientation. Measurements were conducted at the center of each specimen against a white background (CIE L*= 88.81, a*= -4.98, b*= 6.09) and a black backing (CIE L*= 7.61, a*= 0.45, b*= 2.42) by the CIE standard illuminant D65. The lightness L* indicates the variation in shade between black and white, ranging from 0 to 100 (with higher values denoting increased brightness), a* represents the transition in saturation from red to green, and b* denotes the shift from blue to yellow. To quantify TP, the CIELab parameters were employed in the calculation using the Eqs. [37, 38]:

$$\mathrm{TP}={\lbrack{({\mathrm{L*}}_\mathrm{b}\mathrm{-}{\mathrm{L*}}_\mathrm{w}\mathrm{)}}^2+{({\mathrm{a*}}_\mathrm{b}{\mathrm{-a*}}_\mathrm{w})}^2+{({\mathrm{b*}}_\mathrm{b}{\mathrm{b*}}_\mathrm{w})}^2\rbrack}^{1/2}$$

While the CR was calculated using the formula [34, 35]:

$$\mathrm{CR}{\mathrm{(Y}}_\mathrm{b}{\mathrm{/Y}}_\mathrm{w}\mathrm{)}$$

Where b represents the measurement on the black background, w denotes the measurement on the white background, andY represents the test material illuminance.

A higher TP value indicates increased translucency of the substance. A TP score of 100 signifies that the specimen is transparent, whereas a TP value of 0 denotes that the material is opaque [39]. In CR, values may vary from 0 to 1, indicating complete transparency or opacity.

### Specimen preparation for biological testing

#### Cell culture

Human fibroblast cells (HFB4) of 1 × 10^6 cells/mL concentration were purchased from the International Center for Training and Advanced Research (ICTAR-Egypt). Cells were cultured in MEM-E medium, then supplemented with 100 µg/mL of penicillin and 100 µg/mL of streptomycin at 37 °C for 24 h in an atmosphere of 95% air and 5% CO₂ until confluent monolayers were achieved (all reagents were procured from Sigma-Aldrich, Corp., St. Louis, MO, USA). The grown cells were subsequently stored for other applications. A confluent layer of HFB4 cells was dissociated with 0.25% trypsin for 5–10 min. The dissociated cells were reconstituted for growth in culture medium. The concentration was 2 × 10^5 cells per well in 100 µL of culture medium, and plates were incubated for one night. Then, the growth medium was removed, and serum-free medium was introduced to the empty pre-cultured plates [39].

#### Extraction

The GIC was obtained under aseptic conditions within a laminar airflow environment. Each specimen was immersed in a minimum essential medium (MEM) of 10 mg/mL concentration directly and cultured for 7 days at 37 °C, followed by cold centrifugation at 4 °C for 15 min at 4000 rpm (Jouan, KI22-France). The supernatant was filtered through a Millipore sterile filter with a pore size of 0.22 μm.

### Biological properties testing

#### Cytotoxicity test using mtt assay for detection of IC50

To determine cytotoxic concentrations, the nano-GA was diluted in sterile test tubes. Two-fold serial dilutions of the extraction media were prepared using minimum essential medium with Earle’s salts (MEM-E) to achieve twelve distinct concentrations.

The cytotoxicity was assessed by evaluating cell viability at concentrations of 0, 0.5, 1, 2, 4, 8, and 16% of nano-GA included in GIC. Normal human fibroblast (HFB4) cells were cultured in 75 cm² cell culture flasks utilizing MEM-E medium obtained from Gibco, Life Technologies. (Paisley, United Kingdom), supplemented with 10% (v/v) fetal bovine serum (GIBCO™, New York, USA), and incubated in a 5% (v/v) CO₂ incubator at 37 °C. Confluent cells were dissociated utilizing a 0.25% (w/v) trypsin solution and 0.05% (v/v) EDTA (GIBCO™, New York, USA) for 5 min. Cold centrifugation was done to detach cells utilizing a Jouan ki22 refrigerated centrifuge (LABEQUIP LTD, France). Cell pellets were resuspended in the medium of growth. Cells were seeded at a concentration of 2 × 10^5 cells/ml in 96-well cell culture plates (TPP, Switzerland) and kept at 37 °C for 24 h in an incubator (Mini Artic, Jouan, France) to attain confluence. The growth media was removed, and fresh medium with different amounts of the test material extract was introduced for cytotoxicity evaluation via the colorimetric MTT assay. Dead cells were rinsed with phosphate-buffered saline (PBS) and 50 µl of MTT sourced from SERVA, Germany. A stock solution of 0.5 mg/ml was introduced into each well. Following a 4-h incubation, the supernatants were removed, and formazan crystals were solubilized with 50 µl per well of dimethyl sulfoxide (DMSO) and 0.4% acidified isopropanol. Plates were subjected to incubation in the dark. The absorbance was measured at a wavelength of 570 nm utilizing a microplate reader, Elx-800 (Biotek, USA). The percentage of cell viability was determined using the following formula [40]:

$$\mathrm{Cell}\;\mathrm{viability} = (\text{Optical Density of treatment wells}\times{100}) / \text{Optical Density of control wells}$$

Cell viability percentages were graphed against the concentrations of the tested extract. IC50 values for free and encapsulated GA were ascertained using the Masterplex-2010 software application. Cell morphological changes were examined using an inverted microscope (Nikon, Japan).

#### Interleukin-6 (IL-6) and tumor necrosis factor-alpha (TNF-α) Enzyme-Linked Immunosorbent Assay (ELISA)

ELISA for IL-6 and TNF-α was performed according to the manufacturer’s instructions for the Ab178013 Human IL-6 SimpleStep ELISA^®^ Kit and ab181421 Human TNF alpha SimpleStep ELISA^®^ Kit. The IL-6 standard specimen was reconstituted by adding 500 µL Specimen Diluent, and for TNF-α, 1000 µL Specimen NS was added. The mixture was mixed gently. It was held at room temperature for 10 min and mixed gently. The Stock Standard prepared the following dilution series [41].

#### Cell Cycle Analysis (CCA)

HFB4 cells were pre-cultured in 25 cm² cell culture flasks (TPP, Switzerland) and subsequently treated with IC50 values of the test extract for 24 h. The detached and remaining viable cells were collected and carefully fixed for cell cycle analysis with 70% (v/v) methanol (Sigma-Aldrich, UK). Then, it was maintained at 4 °C. Then, cells were resuspended in PBS with 40 µg/ml propidium iodide, 0.1 mg/ml RNase, and 0.1% (v/v) Triton X-100 obtained from Sigma-Aldrich, UK, in a dark environment. Following a 30-min incubation at 37 °C, the cells were examined utilizing a flow cytometer (Becton–Dickinson, San Jose, CA, USA) fitted with an argon ion laser operating at a wavelength of 488 nm. Cell cycle and sub-G1 population were assessed and evaluated as mentioned previously [42].

### Proliferating Cell Nuclear Antigen(*PCNA) ELISA*

The process followed the manufacturer’s instructions for the Human PCNA ELISA kit (NBP3-18219). The process commenced with the preparation of the standard and reagents. Subsequently, washed three times. 100 µL of standards was dispensed into each well, incubated for 2 h at 37℃, followed by three washes. 100 µL of working biotin conjugate antibody was administered. Subsequently, incubated for 1 h at 37℃ and rinsed three times. Incorporate 100 µL of Working Streptavidin-HRP, incubate for 30 min at 37℃, and follow with three washes. Ultimately, 100 µL of substrate solution was introduced. Subsequently, incubate for 15–20 min in the dark at 37℃, then add 50 µL of the stop solution per well.

#### Real-Time PCR (RT-PCR)

Total RNA was isolated from control and treated HFB4 cells using the RNeasy mini kit (Qiagen-USA) according to the manufacturer’s instructions [43]. The concentration and purity of extracted RNA were assessed using a Beckman dual spectrophotometer (Beckman-USA). The expression of apoptosis-related genes was assessed using RT-PCR. Ten nanograms of the isolated RNA from each specimen were utilized for cDNA synthesis employing the high-capacity cDNA reverse transcriptase kit (Thermo Fisher Scientific, USA).

The resultant cDNA was amplified using the SYBR Green I PCR master kit (Thermo Fisher Scientific Inc., Lithuania) and the StepOne apparatus (Applied Biosystems, Thermo Fisher Scientific) for 10 min at 95 °C for enzyme activation. This was succeeded by 40 cycles at 95 °C for 15 s, followed by 55 °C for 20 s, then 72 °C for 30 s during the amplification phase. The expression alterations of the target genes were standardized against the mean critical threshold (CT) values of GAPDH, utilized as a housekeeping gene. The specific primer sequences of genes are shown in Table 2.

**Table 2:** Used specific primer sequences of genes

Bax	F 5’- TCAGGATGCGTCCACCAAGAAG − 3’,
Bax	R 5’- TGTGTCCACGGCGGCAATCATC − 3’
Bcl2	F 5’- ATCGCCCTGTGGATGACTGAGT − 3’,
Bcl2	R 5’- GCCAGGAGAAATCAAACAGAGGC − 3’.
GAPDH	F 5’- GTCTCCTCTGACTTCAACAGCG − 3’
GAPDH	R 5’- ACCACCCTGTTGCTGTAGCCAA − 3’

### Statistical analysis

The distribution analysis was performed to check the normality of the numerical data utilizing the Kolmogorov-Smirnov and Shapiro-Wilk tests. Kruskal-Wallis and Dunn’s tests assessed non-parametric solubility data, while other tests’ data indicated a normal (parametric) distribution. Data were presented as mean and standard deviation (SD) values. Wsp and Wsol, DTS, TP, and CR data were analyzed using a one-way ANOVA, followed by Tukey’s post hoc test. The investigation of shear bond strength utilized two-way ANOVA, followed by comparisons of simple effects based on estimated marginal means, employing the error term from the two-way model. P-values were adjusted for multiple comparisons using the False Discovery Rate (FDR) method. Spearman’s rank-order correlation coefficient was employed for correlation analysis. A two-way ANOVA test was employed to investigate the viability percentage between the two groups and assess the differences among various concentrations within each group. Bonferroni’s post-hoc test was employed for pairwise comparisons when the ANOVA test gave significant results. The Student’s t-test was employed to compare other study variables. The significance level was set at *p* < 0.05 for all analyses. Statistical analysis was conducted using statistical software (IBM SPSS Statistics for Windows: Version 23.0; IBM Corp., NY, USA).

## Results

### Oxidation and nanoformulation of GA

**Fig. 3:**
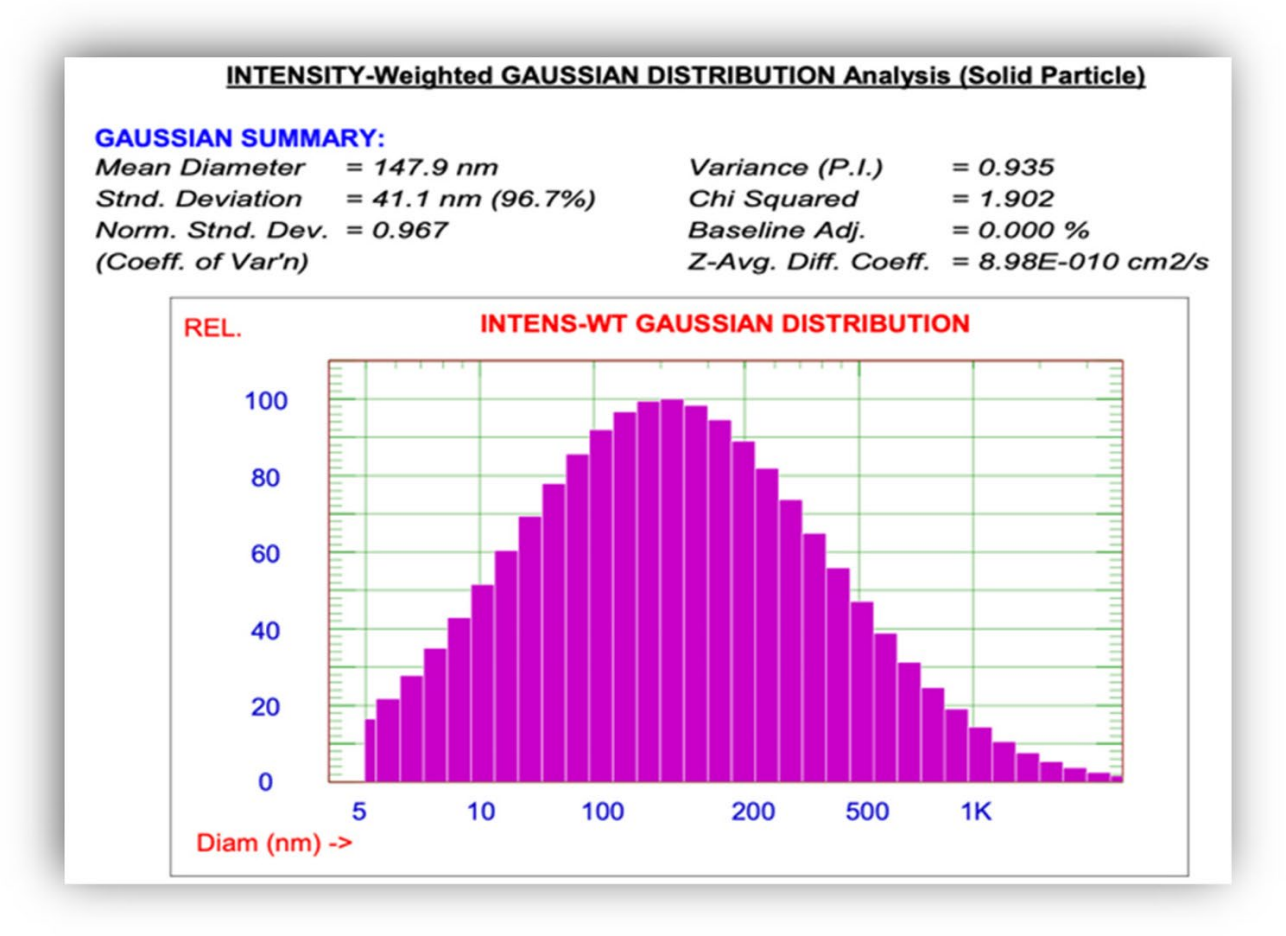
Particle size distribution of oxidized nano GA analyzed by dynamic light scattering (DLS), (mean diameter = 147.9 nm)

Dynamic light scattering (DLS) analysis confirmed the formation of nanoscale particles. The average particle size diameter was 147.9 ± 0.967 nm, and 96.7% of the distribution measured less than 41.1 nm (Fig. 3).

### Physical testing results

#### Film thickness

Pairwise comparisons indicated that the 8.0 wt% group exhibited the highest film thickness among the tested groups, with no significance compared to the 4.0 and 16.0 wt% groups (*P* value < 0.001), as shown in Table 2.

#### Wsp and Wsol

Regarding water solubility, the 2.0 wt% group showed significantly higher values than the 8.0 wt% group. The 0 wt% group had significantly higher values than the 0.5 wt% and 2.0 wt% groups in water sorption (*P* value < 0.001), as noted in Table 3.

**Table 3:** Means and SD of film thickness, Wsp, Wsol, DTS, CR and TP values of different tested GIC groups

Measurement	Mean ± SD								Tests statistic	*p*-value	Effect size			
	0 wt%	0.5 wt%	1.0 wt%	2.0 wt%	4.0 wt%	8.0 wt%	16.0 wt%					PES (95% CI)		Magnitude
Film thickness (µm)	24.00±2.65^C^	25.67±3.51^C^	30.00±2.65^BC^	26.56±7.18^C^	41.89±6.17^AB^	44.11±6.11^A^	43.67±4.04^AB^		10.28	<0.001*		0.815 (0.473:0.839)	Large	
Wsp (%)	8.83±1.72^A^	5.66±0.39^B^	6.58±0.37^AB^	5.90±1.04^B^	7.70±0.58^AB^	8.25±0.69^AB^	7.30±1.27^AB^		4.39	0.011*		0.653 (0.143:0.698)		Large
Wsol (%)	-2.21±2.64^AB^	-4.77±0.31^AB^	-3.19±0.52^AB^	-0.84±0.39^A^	-3.75±0.80^AB^	-5.21±0.43^B^	-2.05±0.72^AB^		14.61	0.024*		0.615 (0.340:0.940)		Large
DTS (MPa)	8.04±1.56^A^	8.64±2.55^A^	8.28±0.70^A^	6.11±0.50^AB^	7.77±1.35^A^	2.37±0.30^B^	3.88±1.15^B^		9.72	<0.001*		0.806 (0.453:0.831)		Large
CR	1.13±0.07^A^	0.93±0.03^B^	0.92±0.04^B^	0.94±0.03^B^	0.96±0.01^B^	0.99±0.05^B^	0.94±0.04^B^		19.65	<0.001*		0.771 (0.593:0.808)		Large
TP	6.43±0.75^D^	9.20±1.55^C^	10.78±0.87^C^	12.85±1.35^B^	4.00±0.68^E^	11.04±0.80^BC^	16.59±1.09^A^		91.68	<0.001*		0.940 (0.889:0.950)		Large

Values with different superscripts within the same horizontal row are significantly different

*PES* Partial Eta Squared

* significant (p<0.05)

### Mechanical and bonding testing results

#### DTS

The 0, 0.5, 1.0, and 4.0 wt% groups had significantly higher diametral tensile strength values than the 8.0 and 16.0 wt% groups (*P* value < 0.001), as shown in Table 3.

#### SBS and mode of failure

Table 4 showed a significant interaction between the nano-GA wt% and the substrate type on SBS. The simple effects comparisons showed that there were differences among different weights. % Modifications for both enamel and dentine were statistically significant. For enamel, the 2.0 wt% group had significantly higher SBS than all other groups except the 0 and 1.0 wt% groups. However, the 0 and 1.0 wt% groups had significantly higher SBS for dentine than all other groups except 2.0 wt%. For the 2.0 wt% group, there was no significant difference in the SBS of both substrates. In contrast, dentine shear bond strength was significantly higher for all other groups than for enamel (*P* value < 0.001).

**Table 4:** Simple effects comparisons for shear bond strength of teated GIC groups

Substrate		Mean ± SD							Tests statistic	p-value	Effect size	
		0 wt%	0.5 wt%	1.0 wt%	2.0 wt%	4.0 wt%	8.0 wt%	16.0 wt%			PES (95% CI)	Magnitude
Enamel		2.66±0.69^AB^	1.55±0.19^B^	3.14±1.02^AB^	4.21±1.47^A^	2.27±0.56^B^	2.20±0.91^B^	1.88±0.65^B^	4.53	<0.001*	0.299 (0.093 to 0.381)	Large
Dentine		7.62±0.94^A^	7.50±1.51^A^	6.07±1.77^AB^	5.55±1.05^B^	4.45±0.43^B^	4.71±0.89^B^	4.56±1.04^B^	10.31	<0.001*	0.493 (0.295 to 0.564)	Large
Test statistic		102.17	66.74	79.11	0.51	19.76	26.10	73.00	4.53	<0.001*	0.299 (0.093 to 0.381)	Large
p-value		<0.001*	<0.001*	<0.001*	0.481ns	<0.001*	<0.001*	<0.001*				
Effect size	PES (95% CI)	0.745 (0.602 0.812)	0.656 (0.479 0.746)	0.693 (0.530 0.774)	0.676 (0.506 0.761)	0.361 (0.150 0.516)	0.427 (0.212 0.570)	0.014 (0.000 0.130)				
	Magnitude	Large	Large	Large	Large	Large	Large	Small				

Values with different superscripts within the same horizontal row are significantly different

*PES* Partial Eta Squared, *ns* Not significant

* significant (*p*<0.05)

**Fig. 4:**
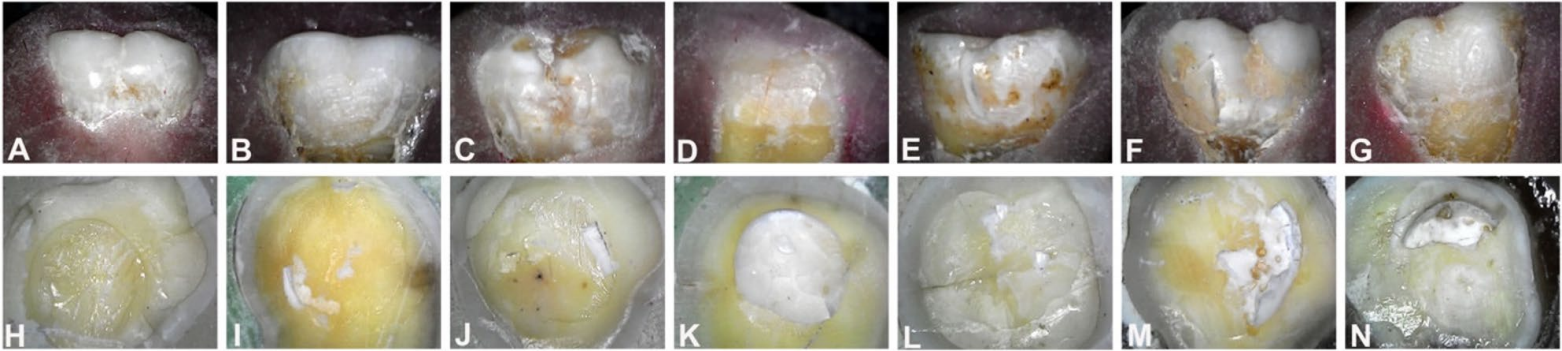
Stereomicroscope images (×35) showing failure modes in enamel (**A**–**G**) and dentin (H–N) after shear bond strength (SBS) testing of GIC specimens with different nano-GA concentrations: 0 wt% (A: adhesive, H: mixed), 0.5 wt% (B: adhesive, I: mixed), 1.0 wt% (C: mixed, J: mixed), 2.0 wt% (D: mixed, K: cohesive), 4.0 wt% (E: mixed, L: mixed), 8.0 wt% (F: adhesive, M: mixed), and 16.0 wt% (G: adhesive, N: mixed)

The failure mode distribution for enamel and dentine demonstrated different failures among the groups. Enamel specimens showed more adhesive failures than dentine, while dentine showed higher mixed failures, as noted (Fig. 4; Table 5).

**Table 5:** Mode of failure of tested GIC groups to enamel and dentine

Substrate	Failure mode	n (%)						
		0%	0.5%	1.0%	2.0%	4.0%	8.0%	16.0%
Enamel	Adhesive	6 (60%)	4 (40%)	7 (70%)	8 (80%)	7 (70%)	2 (20%)	4 (40%)
	Cohesive	0 (0%)	0 (0%)	0 (0%)	0 (0%)	0 (0%)	0 (0%)	0 (0%)
	Mixed	4 (40%)	6 (60%)	3 (30%)	2 (20%)	3 (30%)	8 (80%)	6 (60%)
Dentine	Adhesive	0 (0%)	2 (20%)	0 (0%)	0 (0%)	0 (0%)	0 (0%)	3 (30%)
	Cohesive	3 (30%)	3 (30%)	0 (0%)	3 (30%)	2 (20%)	0 (0%)	2 (20%)
	Mixed	7 (70%)	5 (50%)	10 (100%)	7 (70%)	8 (80%)	10 (100%)	5 (50%)

### Optical testing results

#### TP and CR

Even though the GIC specimens with varying weight percentages of nano-GA showed a discernible rise in discoloration upon visual inspection (Fig. 1), the amount of discoloration was slightly affected by adding 0.5, 1.0, and 2.0 wt% of nano-GA to GIC.

The color parameters (L*, a*, b*) of the experimental GICs showed statistically significant differences among all groups under both white and black backgrounds (*p* < 0.001, Table 5). The mean L* values decreased progressively with increasing nano–Gum Arabic concentration, indicating a gradual darkening of the material. In contrast, the a* and b* coordinates increased with higher concentrations, reflecting a shift toward more reddish and yellowish tones. The control group (0%) exhibited the highest L* and the lowest a* and b* values, whereas the 16% group showed the lowest L* and the highest a* and b* values. The background color did not produce noticeable differences in any of the parameters, suggesting consistent optical behavior across both measurement conditions, as shown in Table 6.

For the TP, the 16.0 wt% group had significantly the highest values, while the 0 wt% group had the lowest values. The 2.0% group showed significantly higher values than 0, 0.5, 1.0, and 4.0 wt%. Additionally, the 0.5 and 1.0 wt% groups had significantly higher values than the 0 wt% group, while the 0 wt% group had significantly higher values than the 4.0 wt% group. Regarding CR, the control group exhibited significantly the highest values, as shown in Table 2. A moderate negative correlation between CR and TP was noted [rs = -0.389 (95% CI -0.619 to -0.096), *p* = 0.011].

**Table 6:** Means and SD of CIELab color coordinates of tested GIC groups after 7 days in black and white background

Group	Mean ± SD					
	White background			Black background		
	L*	a*	b*	L*	a*	b*
0%	118.40 ± 0.57^A^	-2.03 ± 0.22^C^	-3.57 ± 0.39^D^	118.40 ± 0.57^A^	-2.03 ± 0.22^C^	-3.57 ± 0.39^D^
0.5%	95.45 ± 1.17^C^	-2.60 ± 0.33^C^	-3.32 ± 1.83^CD^	95.45 ± 1.17^C^	-2.60 ± 0.33^C^	-3.32 ± 1.83^CD^
1.0%	94.03 ± 1.43^C^	-1.77 ± 0.54^C^	-1.68 ± 0.99^CD^	94.03 ± 1.43^C^	-1.77 ± 0.54^C^	-1.68 ± 0.99^CD^
2.0%	99.42 ± 0.59^B^	-1.43 ± 0.68^C^	-0.63 ± 1.93^C^	99.42 ± 0.59^B^	-1.43 ± 0.68^C^	-0.63 ± 1.93^C^
4.0%	92.75 ± 5.02^C^	0.62 ± 1.18^B^	5.22 ± 0.97^B^	92.75 ± 5.02^C^	0.62 ± 1.18^B^	5.22 ± 0.97^B^
8.0%	82.57 ± 1.37^D^	1.22 ± 1.32^B^	6.22 ± 2.18^B^	82.57 ± 1.37^D^	1.22 ± 1.32^B^	6.22 ± 2.18^B^
16.0%	70.23 ± 0.92^E^	4.37 ± 1.81^A^	13.15 ± 1.86^A^	70.23 ± 0.92^E^	4.37 ± 1.81^A^	13.15 ± 1.86^A^
Test statistic	37.74	36.59	36.36	33.81	30.19	32.52
p-value	< 0.001*	< 0.001*	< 0.001*	< 0.001*	< 0.001*	< 0.001*
PES [H] (95% CI)	0.91 (0.90:0.95)	0.87 (0.84:0.94)	0.87 (0.83:0.95)	0.79 (0.77:0.94)	0.69 (0.62:0.87)	0.76 (0.72:0.95)
Magnitude	Large	Large	Large	Large	Large	Large

### Biological results

#### Cell viability through MTT assay

**Fig. 5:**
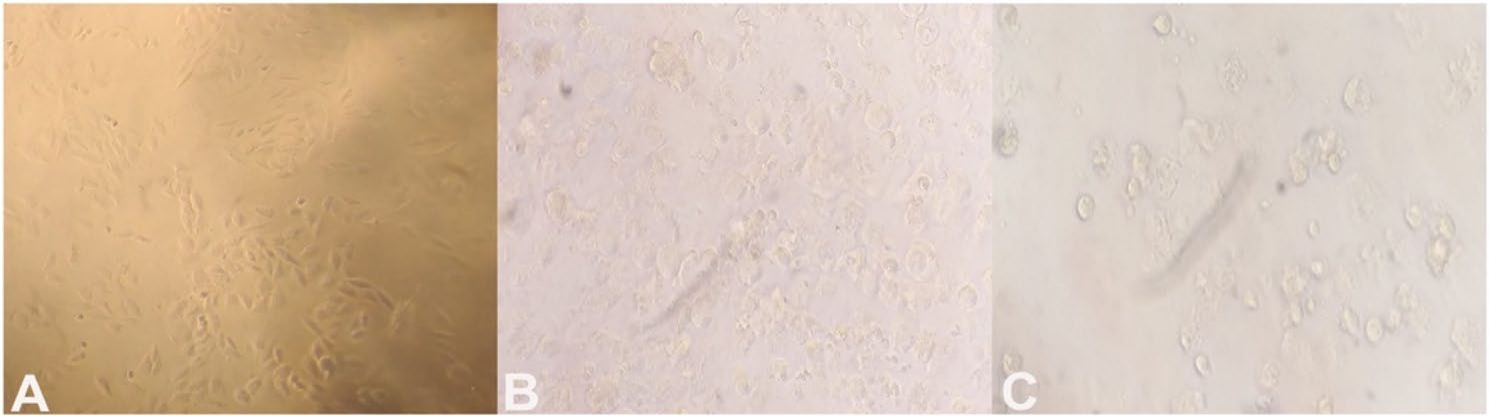
Morphological alterations of cells were analyzed using an inverted microscope (Scale bar 100μm) **A** showed control group, cells appears spindle fusiform in shape, **B** 8% concentration of nano-GA, **C** 16% concentration of nano-GA

After IC-50 was detected, morphological alterations of cells against 8.0 wt% and 16.0 wt% concentrations of nano-GA were examined using an inverted microscope (Nikon, Japan) (Fig. 5). This revealed a change in the standard form of the spindle fibroblast to more rounded, irregular cells.

#### Viability and detection of IC50

Viability% is the concentration required to induce cytotoxicity in 50% of the tested cells. The concentrations were gradually increased till they reached the lethal dose. Regarding the 8.0 wt % group, there was a statistically significant difference between the viability % values of different concentrations. Pairwise comparisons between the concentrations revealed that the concentration of 0.2 showed the statistically significantly highest mean viability %. There was no statistically significant difference between concentrations of (25), (12.5), (6.25), (3.12), (1.56), (0.78), and (0.4); all showed statistically significantly lower values. Concentration of (50) showed statistically significantly lower mean viability %, followed by concentration of (100).

**Fig. 6:**
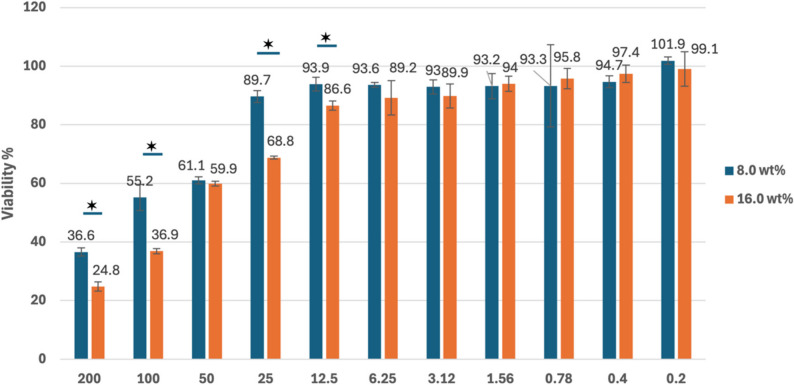
Bar chart showing the mean cell viability (%) for the 8.0 wt% and 16.0 wt% groups across different concentrations (200–0.2 µg/mL). Error bars represent mean ± standard deviation (SD). Sample size:*n* = 6 per group. Significant differences between the two groups at each concentration are indicated by asterisks (*p < 0.001)

Concentration of (200) showed the statistically significantly lowest mean viability %. Meanwhile, for group (16%), there was a statistically significant difference between viability % values of different concentrations. Pairwise comparisons between the concentrations revealed that there was no statistically significant difference between concentrations of (0.2), (0.4), (0.78), and (0.156); all showed the statistically significantly highest mean viability %. There was no statistically significant difference between concentrations of (12.5), (6.25), and (3.12); all showed statistically significantly lower values. Concentration of (25) showed statistically significantly lower mean viability %, followed by concentration of (50), then concentration of (100). Concentration of 200 showed the statistically significantly lowest mean viability percentage (Fig. 6).

**Fig. 7:**
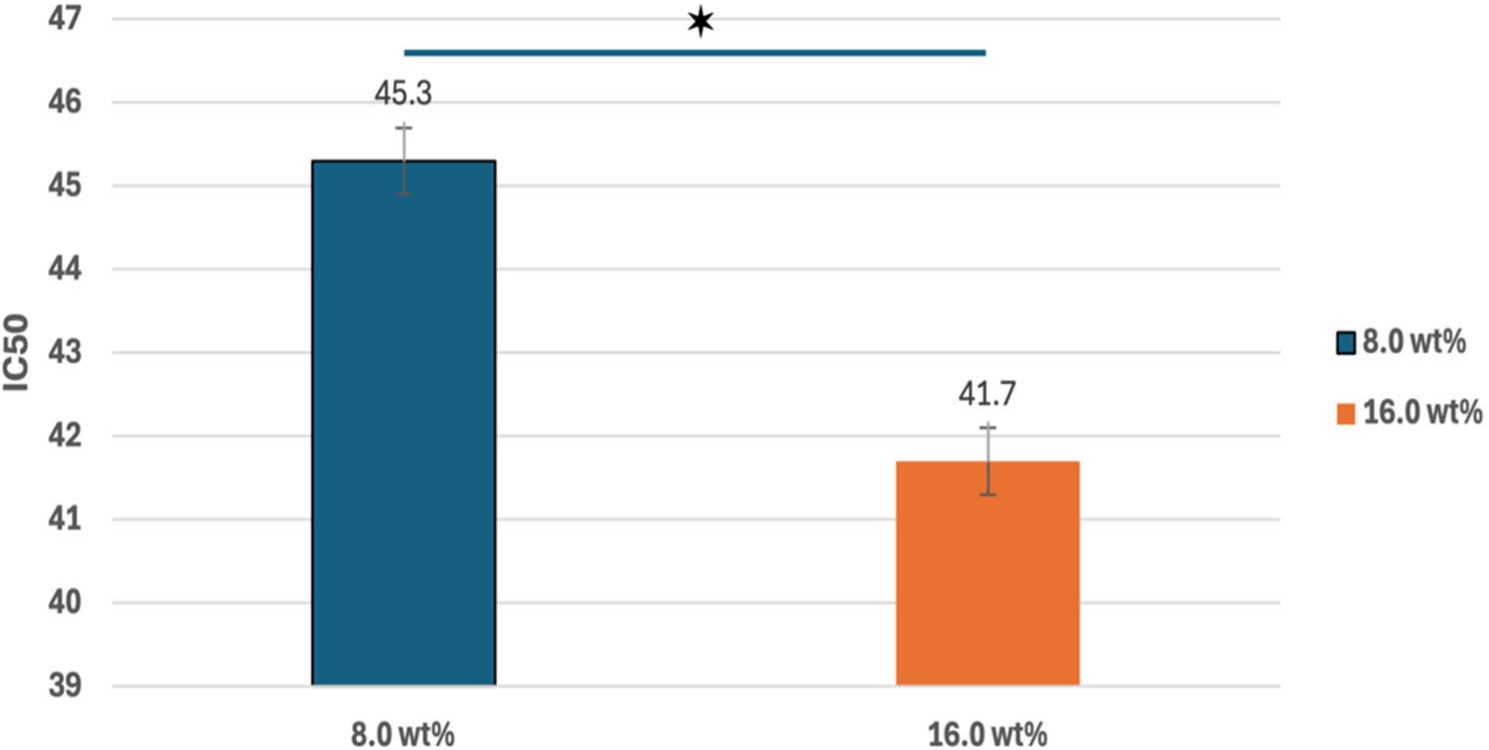
Comparison of IC₅₀ values between the 8.0 wt% and 16.0 wt% groups. Bars represent the mean IC50, and error bars indicate mean ± standard deviation (SD). Sample size:*n* = 6 per group. Significant difference between the two groups are indicated by asterisk (*p < 0.001)

Concentrations started from zero to 6, showing a harmless material without cytotoxicity. The results showed that group (8.0 wt%) had a statistically significantly higher mean IC50 than group (16.0 wt%), *P* value < 0.001* (Fig. 7).

### RT-PCR (BAX & Bcl-2)

The results of the two primers with the Bax primer revealed that group (8.0 wt%) showed statistically significantly lower mean fold change than group (16.0 wt%). With the B-cl2 primer, group (8.0 wt%) showed statistically significantly higher mean fold change than the group (16.0 wt%). Regarding Bax/Bcl-2 ratio: The Group (8.0 wt %) showed a statistically significantly lower mean Bax/Bcl-2 ratio than the group (16.0 wt%), *p* value: 0.001.

### TNF-α, IL-6, and PCNA (pg/ml)

Group (8.0 wt%) had a statistically significantly lower mean TNF-α concentration than group (16.0 wt%). Considering IL-6, a statistically significant difference between the groups was recorded. Pairwise comparisons revealed that the control group showed the statistically significantly highest mean IL-6. Group (8.0 wt%) showed a statistically significantly lower mean value, and group (16.0 wt%) showed the statistically significantly lowest mean IL-6 with *p* value < 0.001. For PCNA, a statistically significant difference was revealed between the groups. Pairwise comparisons revealed that the control group showed the statistically significantly highest mean PCNA. The group (8.0 wt%) showed a statistically significantly lower mean value. Group (16.0 wt%) showed the statistically significantly lowest mean PCNA, with a p-value < 0.001.

### CCA (DNA content)

**Fig. 8:**
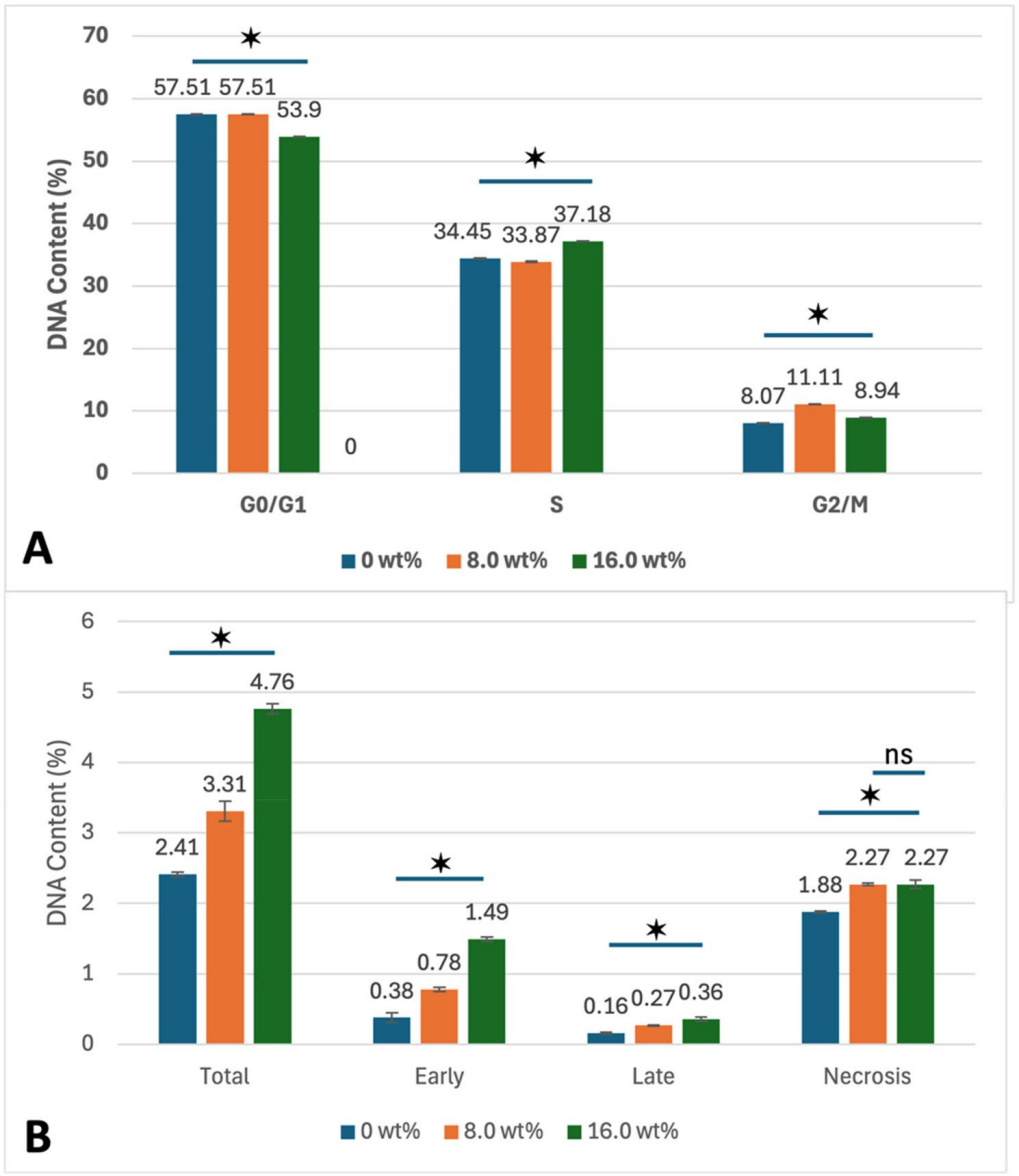
Bar charts illustrating (**A**) the cell-cycle distribution (G0/G1, S, and G2/M phases) and (**B**) the percentages of total apoptosis, early apoptosis, late apoptosis, and necrosis in the 0 wt%, 8.0 wt%, and 16.0 wt% groups. Bars represent the mean DNA content (%), and error bars indicate mean ± standard deviation (SD). Sample size: *n* = 6 per group. Significant differences between groups within each cell-cycle phase are marked with asterisks (*p < 0.001), and non-significant differences are denoted as (ns)

G0/G1 phase (%): There was a statistically significant difference between the groups. Pairwise comparisons revealed that the control group statistically showed the highest mean G0/G1. (8.0 wt %) The group showed a statistically significantly lower mean value. The group of 16% showed the statistically significantly lowest mean G0/G1, with a p-value < 0.001. In the S phase, a statistically significant difference between the groups was revealed. Pairwise comparisons revealed that the group (16.0 wt%) showed the statistically significantly highest mean S. Statistically, the mean value was significantly lower for the control group. The group of 8% showed the statistically significantly lowest mean S, with a p-value < 0.001*. G2/M phase (%): A statistically significant difference between the groups was shown. Pairwise comparisons revealed that the group (8.0 wt%) showed the statistically significantly highest mean G2/M. The group of 16.0 wt% showed a statistically significantly lower mean value. The control group showed the statistically significantly lowest mean G2/M, with a p-value < 0.001 (Fig. 8a).

### Apoptosis

Regarding total apoptosis, there was a statistically significant difference between the groups. Pairwise comparisons revealed that the group (16.0 wt%) showed the statistically significantly highest mean total apoptosis. The group (8.0 wt%) was statistically significantly lower in mean value. The control group showed the statistically significantly lowest mean total apoptosis. In early apoptosis, there was a statistically significant difference between the groups. Pairwise comparisons revealed that (16.0 wt%) showed the statistically significantly highest mean early apoptosis. (8.0 wt%) showed a statistically significantly lower mean value. The control group showed the statistically significantly lowest mean early apoptosis. Considering late apoptosis, there was a statistically significant difference between the groups. Pairwise comparisons revealed that the group (16.0 wt%) showed the statistically significantly highest mean late apoptosis. The group of 8.0 wt% showed a statistically significantly lower mean value. The control group showed the statistically significantly lowest mean late apoptosis. For total necrosis, there was a statistically significant difference between the groups. There was no statistically significant difference between groups of (8.0 wt%) and (16.0 wt%) with pairwise comparisons. Both showed significantly higher mean necrosis than the control group (Fig. 8b; Table 7).

**Table 7:** Descriptive statistics and results of one-way ANOVA test for comparison between DNA contents in the three tested groups

DNA Content	0 wt%		8.0 wt%		16.0 wt%		*P*-value	Effect size (Eta squared)
	Mean	SD	Mean	SD	Mean	SD		
G0/G1 (%)	57.51 ^A^	0.07	55.04 ^B^	0.05	53.9 ^C^	0.06	< 0.001*	0.999
S (%)	34.45 ^B^	0.03	33.87 ^C^	0.08	37.18 ^A^	0.04	< 0.001*	0.999
G2/M (%)	8.07 ^C^	0.04	11.11 ^A^	0.03	8.94 ^B^	0.08	< 0.001*	0.999

G0: Resting phase. G1: Gap1, S: Synthesis, M: Mitosis, G2: Gap2

*: Significant at *P* ≤ 0.05

## Discussion

Oxidation of GA was performed to introduce carboxyl and other acidic functional groups on the polysaccharide chains. These groups can interact with the calcium and aluminum ions in the GIC powder, potentially enhancing crosslinking and mechanical properties [28, 44].

In the current study, the incorporation of 0.5, 1.0, and 2.0 wt% nano-GA in GIC has had a relatively minor influence on the discoloration of GIC. The color change in these GA-GIC formulations was expected to be considered acceptable for dental restorations. However, a similar observation was reported in 0.5 and 1% micro GA-GIC [45].

The first hypothesis was partially rejected, as nano-GA incorporation significantly affected the film thickness of GIC. According to ISO 9917-1:2007, luting cements should not exceed a film thickness of 25 μm; however, all nano-GA-modified groups surpassed this limit, while the control group remained within the acceptable range. The progressive increase in film thickness with higher nano-GA concentrations suggests that nanoparticle addition altered the material’s rheology by increasing viscosity and restricting flow. These findings are consistent with previous reports that filler or polymer reinforcement can elevate GIC film thickness [28, 45]. The difference with studies using micro-GA [32] may be attributed to differences in particle size, surface reactivity, and dispersion behavior, as nanoscale particles exhibit greater surface area and stronger intermolecular interactions, leading to reduced flowability and thicker films.

Nano-GA incorporation showed limited influence on the water solubility and sorption behavior of GIC. Negative mean solubility values observed across all groups likely resulted from partial dehydration rather than actual material loss, reflecting water retention within the maturing cement matrix [46–48]. This is consistent with the prolonged acid-base reaction of GICs, during which bound and absorbed water becomes an integral component of the structure [49]. The highest negative solubility values were recorded for the 8.0 wt% group, while the 2.0 wt% group exhibited the lowest, indicating non-uniform water incorporation and partial entrapment within the matrix-filler interfaces.

The first hypothesis was partially accepted as all formulations showed water sorption upon immersion, with the control group showing the highest uptake. Two main theories explain water diffusion in polymeric materials. The free volume theory proposes that water diffuses through microvoids without interacting with polar groups, while the interaction theory suggests that water binds to hydrophilic sites during diffusion. The latter occurs when diffusion and equilibrium water uptake show a negative correlation [50].The reduced sorption observed in the 0.5 wt% and 2.0 wt% nano-GA groups suggests that optimally dispersed nanoparticles may occupy microvoids and enhance matrix crosslinking, thereby limiting water diffusion. At higher concentrations, however, GA aggregation and interference with cement setting likely negate this effect, producing sorption values comparable to the control. The absence of significant differences among modified groups supports that nano-GA did not markedly alter the chemical structure of the base GIC [50]. Nonetheless, reports of increased sorption in GA-modified systems have been linked to the hydrophilic character of GA, a polysaccharide rich in hydroxyl (-OH) groups capable of hydrogen bonding with water molecules [51].

In this study, a USB digital microscope was employed to evaluate the failure modes of SBS specimens. Although Although scanning electron microscopy (SEM) offers higher magnification and more detailed imaging, the digital microscope was chosen for its practicality, minimal preparation, and adequate resolution to classify failures as adhesive, cohesive, or mixed. Previous studies confirmed its reliability in dental bonding analysis, showing comparable results to SEM for general failure classification [52, 53].

The second hypothesis was partially rejected, as the DTS values of the modified GICs (0.5-4.0 wt%) were statistically comparable to those of the unmodified control, indicating that nano-GA incorporation did not markedly alter the structural integrity of the cement matrix. The minor enhancement observed at lower concentrations may be attributed to nano-GA particles filling microvoids formed during mixing, thereby improving matrix densification and reducing internal defects [54, 55]. Additionally, the nanoparticles may introduce supplementary reactive sites for polyacrylic acid, facilitating localized ionic and hydrogen bonding and contributing to limited structural reinforcement [56, 57]. The presence of nanoscale fillers may also promote a more ductile interphase, attenuating crack propagation and improving energy dissipation under stress [58, 59]. Moreover, GA’s viscoelastic nature may enable partial absorption of deformation energy, enhancing the material’s resistance to brittle fracture [60]. Conversely, excessive nano-GA content (8.0 and 16.0 wt%) resulted in a reduction in mechanical strength, likely due to nanoparticle agglomeration and disruption of the acid-base setting reaction, which generate weak interfacial zones and porosity within the matrix [61, 62].

The second hypothesis was partially accepted, as nano-GA incorporation significantly influenced GIC bonding performance. The 2.0 wt% group showed the highest enamel bond strength, while higher concentrations reduced adhesion, likely due to interference with ionic interactions between polyacid (COO⁻) and calcium (Ca²⁺) ions in enamel and dentin. At 0.5–1.0 wt%, dentin bonding remained comparable to the control, indicating that low nano-GA levels do not hinder chemical adhesion. Except for 16.0 wt%, dentin bond strength exceeded enamel, consistent with the dual bonding mechanism involving ionic exchange and micromechanical interlocking [63]. The reduced bond strength to dentin at lower nano-GA levels, despite stable enamel adhesion, may be attributed to dentin’s heterogeneous and hydrated nature, which impedes cement penetration and ionic bonding. In contrast, enamel’s homogeneous, mineralized structure favors consistent adhesion. Increasing nano-GA content may enhance filler–matrix interaction and reinforce the cement network, partially compensating for dentin’s structural limitations.

Fracture modes in enamel and dentin were assessed using optical microscopy. Although SEM imaging would provide a more precise evaluation, the current study focused primarily on bond strength. Failure analysis revealed predominantly cohesive and mixed failures, with adhesive failures more frequent at the enamel interface, reflecting weaker interfacial adhesion relative to cohesive strength [64, 65]. Cohesive failures at the dentin interface indicate intrinsic material limitations, with mixed cohesive dominance aligning with reports that the GI-tooth bond often exceeds internal strength [56, 64]. Stress concentration during SBS testing may have contributed to cohesive failures [65], as confirmed by SEM observations in previous studies [66–68].

The third hypothesis was rejected, as nano-GA addition significantly affected the TP of GICs. The 16.0 wt% group showed the highest TP, followed by 8.0 wt% and 2.0 wt% groups, indicating enhanced translucency with increasing nano-GA content. However, excessive translucency may yield an undesired grayish appearance intraorally, especially in extensive Class III restorations. In contrast, the 0 wt% and 4.0 wt% groups demonstrated very low TP values, reflecting excessive opacity unsuitable for esthetic applications. The observed inverse relationship between TP and CR agrees with previous findings for GIC-based materials [69].

Colorimetric data further revealed that increasing nano-GA concentrations reduced L* values (darkening effect) and elevated a* and b* values, shifting the hue toward reddish-yellow tones. These optical changes are likely due to the inherent color and refractive mismatch of oxidized GA nanoparticles, which increase light scattering within the matrix, consistent with prior studies on bio-based nanofillers [32]. Background color had no significant effect, indicating stable optical response. Overall, low nano-GA levels (≤ 2.0 wt%) maintained acceptable translucency and color balance, whereas higher concentrations negatively impacted esthetic performance.

The selection of an appropriate cell line is critical for accurate evaluation of GIC biocompatibility in in vitro cytotoxicity studies. Permanent cell lines are generally preferred over primary cultures due to their morphological and physiological uniformity, reproducibility, and stability, despite slightly lower clinical relevance [70]. Accordingly, this study employed permanent human fibroblasts, consistent with Kolado et al. [26] and ISO 10,993 guidelines, which recommend established cell lines for standardized in vitro cytotoxicity assessment [71]. Cytotoxicity constitutes a primary determinant of material safety, and among available assays, the ISO-endorsed MTT test was utilized to quantify succinate dehydrogenase activity, reflecting cellular viability [72]. Furthermore, evaluation of cell cycle progression and apoptosis provides additional insight into cytocompatibility, as perturbations in these processes may indicate potential genotoxicity or carcinogenic risk [73].

In the present study, higher nano-GA concentrations (8.0 and 16.0 wt%) induced increased G0/G1 phase arrest and total apoptosis, particularly at 16.0 wt%, demonstrating a concentration-dependent cytotoxic response. Accordingly, the fourth hypothesis was partially accepted. Comparable observations were reported by Ersahan et al. [74] and Diemer et al. [75], who noted slight reductions in cell viability in conventional and self-adhesive cements.

This study also represents a pioneering assessment of the genotoxic potential of nano-GA-modified GICs via RT-PCR analysis of apoptotic markers *BAX* and *BCL-2*, which were downregulated at 8.0 and 16.0 wt%, suggesting activation of pro-apoptotic and inflammatory pathways at higher concentrations. Although GA exhibits antioxidant and chemoprotective properties due to its amino acid composition [76], excessive nanoparticle incorporation may compromise DNA integrity, consistent with previous reports [77–79].

Conventional GICs generally exhibit lower cytotoxicity than resin-modified or ceramic-reinforced variants [80–83]. However, fluoride release from GICs may induce mild apoptotic effects [84], and the low initial setting pH of luting formulations has been implicated in early cytotoxic responses by promoting transient cellular stress [85]. Incorporation of biocompatible additives such as nano-GA therefore aims to enhance mechanical performance while preserving or minimally influencing the biological safety of the material.Inflammatory marker analysis revealed elevated TNF-α, IL-6, and PCNA levels at 16.0 wt% nano-GA, indicating increased inflammatory potential. IL-6, a multifunctional cytokine, regulates both apoptosis resistance and cell differentiation [86]. Consistently, Ahmed et al. [87] reported that conventional Medicem GIC demonstrates favorable biocompatibility compared with resin-based cements.

Finally, it should be emphasized that in vitro findings cannot be directly extrapolated to clinical conditions, as residual dentin thickness in restorative procedures provides a natural diffusion barrier and buffering capacity that may attenuate the cytotoxic effects of luting materials [88, 89].

Comparison of the present findings with previous studies is limited by the scarcity of data and methodological variations concerning nano-GA incorporation into GICs. The experiments were conducted under controlled in vitro conditions that may not fully replicate the clinical environment, where factors such as saliva, temperature fluctuations, and host immune responses could influence material performance and cytotoxicity. Furthermore, artificial aging was not applied, restricting the assessment of long-term stability and durability. Future studies should include preliminary optimization to identify the most effective nano-GA concentrations before comprehensive evaluation. Investigations into nanoparticle size, degree of oxidation, and interactions with glass fillers are essential to better understand their effects on the physical, mechanical, and biological behavior of GICs. Complementary characterization using FTIR and TEM is recommended to elucidate the chemical structure and morphology of the modified materials. Long-term studies assessing wear resistance, fluoride release, and bonding durability under thermal and mechanical cycling are warranted. Future studies may also incorporate SEM to enhance the analysis of fracture patterns, along with cytocompatibility testing using different cell lines, to further strengthen the clinical relevance of the findings.

## Conclusions

Incorporating nano-GA into conventional GIC improved its physical, mechanical, adhesive, optical, and biological properties in a concentration-dependent manner. Optimal performance was achieved at 0.5-2.0 wt% nano-GA, enhancing strength, adhesion, and stability without affecting translucency or biocompatibility. Nano-GA up to 4.0 wt% was biocompatible, while higher concentrations induced cytotoxic effects. Thus, nano-GA is a promising additive for improving GIC performance at optimized concentrations.

## Data Availability

The datasets used and/or analysed during the current study are available from the corresponding author on reasonable request.
